# Automatic Identification of Motion Artifacts in EHG Recording for Robust Analysis of Uterine Contractions

**DOI:** 10.1155/2014/470786

**Published:** 2014-01-09

**Authors:** Yiyao Ye-Lin, Javier Garcia-Casado, Gema Prats-Boluda, José Alberola-Rubio, Alfredo Perales

**Affiliations:** ^1^Grupo de Bioelectrónica (I3BH), Universitat Politècnica de València, Camino de Vera s/n Ed.8B, 46022 Valencia, Spain; ^2^Servicio de Obstetricia, H. U. La Fe, Valencia, Spain

## Abstract

Electrohysterography (EHG) is a noninvasive technique for monitoring uterine electrical activity. However, the presence of artifacts in the EHG signal may give rise to erroneous interpretations and make it difficult to extract useful information from these recordings. The aim of this work was to develop an automatic system of segmenting EHG recordings that distinguishes between uterine contractions and artifacts. Firstly, the segmentation is performed using an algorithm that generates the TOCO-like signal derived from the EHG and detects windows with significant changes in amplitude. After that, these segments are classified in two groups: artifacted and nonartifacted signals. To develop a classifier, a total of eleven spectral, temporal, and nonlinear features were calculated from EHG signal windows from 12 women in the first stage of labor that had previously been classified by experts. The combination of characteristics that led to the highest degree of accuracy in detecting artifacts was then determined. The results showed that it is possible to obtain automatic detection of motion artifacts in segmented EHG recordings with a precision of 92.2% using only seven features. The proposed algorithm and classifier together compose a useful tool for analyzing EHG signals and would help to promote clinical applications of this technique.

## 1. Introduction

Monitoring uterine contractions is commonly used to estimate the time of an approaching labour. In spite of the fact that intrauterine pressure (IUP) is regarded as the *gold standard* in monitoring these contractions, its use in clinical practice is limited since it requires rupturing the membranes to place a catheter inside the uterus. This not only leads to delivery but may also increase the risk of intrapartum infection [1]. Hospitals often use a pressure transducer (TOCOdynamometer or TOCO) placed on the mother's abdomen for basic noninvasive monitoring of uterine activity, thus obtaining the frequency and duration of contractions. However, the TOCO is not a reliable technique, as the measurements obtained are by no means precise and depend to a large extent on the subjective criteria of the operator [2–4]. Neither do they provide much additional information on the efficiency of contractions in order to decide whether parturition is near. However, in spite of these disadvantages, the technique is widely used in maternity clinics due to its non-invasive nature.

The electrohysterogram (EHG) is the recording of uterine electrical activity from the abdominal surface. Earlier studies have shown that the EHG signal is synchronized in time with the electrical signal generated by the myometrial smooth muscle, which is also related in time with uterine contractions in all animal species, including humans [2, 5, 6]. In addition, the EHG also provides relevant information for assessing the efficiency of contractions, due to the fact that as pregnancy advances and the time of birth approaches uterine electrical activity undergoes changes which are reflected in EHG signals temporal and spectral characteristics [2, 3, 5, 7, 8]. Moreover, recent studies have shown that conduction velocity and direction are associated with the contractions efficiency [8–11].

However, due to the difficulties involved in interpreting the information contained in EHG recordings, this non-invasive technique is still not used in clinical practice. In order to promote its clinical application different methods have been applied to extract from EHG record a signal which is similar to pressure recordings (TOCO-like signal), with which clinical staff are familiar [3, 12–16], and algorithms have been developed to allow contractions to be detected automatically in the TOCO-like signals [12, 14, 16]. The main problem associated with the extensive application of these algorithms lies in the fact that EHG recordings contain not only uterine electrical activity but also a series of physiological interference elements (maternal and fetal ECG, abdominal muscle activity, and baseline fluctuations) and motion artifacts [3, 12, 17]. The presence of the latter phenomena can completely distort the spectral power density [18–21] which could lead to misinterpretation of the results. In addition, the presence of such artifacts makes the automatic identification of contractions based on TOCO-like signals generated from the EHG signal extremely difficult [12]. This is the reason why so many authors consider it necessary to have the recordings segmented manually prior to data analysis by experts in identifying signal windows containing contractions [7, 22–24]. This is a crucial task as it has repercussions on the information that may subsequently be extracted. However, it is also laborious and costly, not to mention the fact that the results are partially dependent on the subjective criteria of the operator. The aim of this work was therefore to develop a tool that would provide automatic segmentation of EHG recordings while distinguishing between uterine contractions and artifacts, to promote the future clinical use of this non-invasive technique for dynamic uterine monitoring and predicting premature births.

## 2. Materials and Methods

### 2.1. Data Acquisition

Twelve recording sessions were carried out at the *Hospital Universitario y Politécnico la Fe* in Valencia (Spain) on twelve healthy women in the first stages of labor having uneventful singleton pregnancies. Estimated gestational period was 37–41 weeks. The study adheres to the Declaration of Helsinki and was approved by the Ethical Committee of the hospital. All the volunteers were informed of the nature of the study, briefed on the recording protocol, and signed the consent form. The duration of the sessions was between 30 minutes and two hours. Patients were asked to report about fetal movements they could identify. Maternal movements were written down by the examiner.

**Figure 1:**
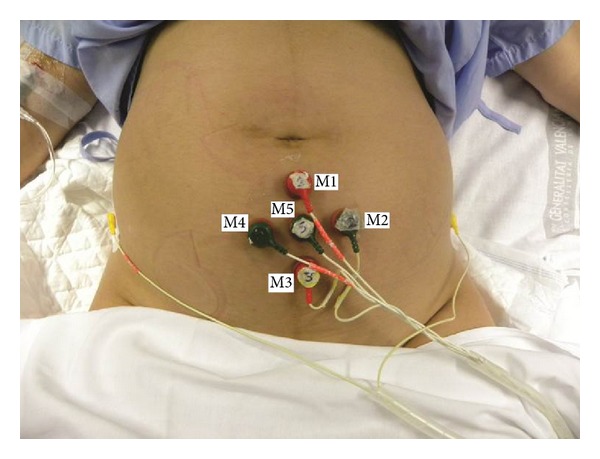
Configuration of contact electrodes for EHG recording.

The subjects were prepared by applying abrasive paste to the skin surface to reduce electrode contact impedance. In each session, 5 monopolar EHG signals were acquired through 5 unshielded Ag/AgCl electrodes with 8 mm in recording diameter placed in the form of a cross in the subumbilical zone, as shown in Figure 1. This arrangement was chosen since the best EHG signal/noise ratio is obtained close to the abdominal vertical midline, especially in the region immediately below the umbilicus [15]. Similar to other studies interelectrode distance was 25 mm [7, 8, 25] and the reference electrode was placed on the subjects' right hip [15, 24]. A third electrode was placed on the subjects' left hip and it was connected to the commercial bioamplifiers' ground terminal. All the monopolar EHG signals were amplified, analog bandpass filtered between 0.05 and 35 Hz (Biopac ECG100C), and acquired at a sampling frequency of 500 Hz.

At the same time a TOCOdynamometer was positioned on the abdominal surface together with an intrauterine pressure catheter (ACCU-Trace) to obtain the TOCO and IUP readings, respectively. The signals were conditioned in a commercial maternal/fetal monitor (Corometrics 170 Series, GE Medical Systems) and acquired at a sampling frequency of 4 Hz. All data were simultaneously stored in a PC for subsequent analysis.

### 2.2. Signal Preprocessing

Since EHG signal energy basically ranges from 0.1 to 3–5 Hz [5, 15, 26], a 5th order Butterworth bandpass digital filter between 0.1 and 4 Hz was used to eliminate undesirable components. Then monopolar EHG signals were downsampled at 20 Hz to reduce the computational cost of the data analysis. This sampling frequency is enough to compute the spectral parameters later described and showed no significant effects in the distribution of the nonlinear parameters studied in the next section. In this study, only the 4 bipolar EHG signals obtained from monopolar recordings were analyzed, since this configuration largely reduces the amount of interference present in monopolar EHG recordings [5, 15, 24, 27]:

(1) $$\begin{array}{c} B_{1}=M_{1}-M_{5};\;\;B_{2}=M_{5}-M_{3};\\ B_{3}=M_{4}-M_{5};\;\;B_{4}=M_{5}-M_{2}, \end{array}$$

where *M*_*i*_ are the preprocessed EHG signals acquired by electrode *i* (*i* = 1,…, 5) and *B*_*j*_ (*j* = 1,…, 4) are the estimated bipolar signals.

As has been mentioned above, identifying uterine contractions in EHG recordings is usually performed by means of the TOCO-like signals derived from them [12, 14]. In the present study, in order to exclude from the analysis most of the artifact components due to motion, respiration, and cardiac electrical signals, only frequency components in the 0.34–1 Hz (“uterine specific” range) [3, 22, 28] were used for generating the TOCO-like signal of the four bipolar signals. Concretely, two TOCO-like signals from bandpass filtered EHG bipolar signal were obtained by calculating the RMS value [12, 29] and the unnormalized first statistical moment of the frequency spectrum [15] of 30 s moving windows displaced every 0.25 s.

The TOCO-like signal segments with an amplitude significantly different to that of the baseline were then identified following a criteria similar to other authors' proposals for automatic detection of contractions [12, 22]. The baseline activity of each TOCO-like signal was obtained with a 4-minute moving window displaced every 0.25 s, ordering the TOCO-like signal from highest to lowest values and calculating the average of the lowest 10% of values. The signal segments with an amplitude significantly different to that of the baseline were then identified when the TOCO-like signal amplitude remained at >2*x*, the mean baseline activity, and at >25%, signal amplitude of each window for more than 30 s. A rise in amplitude in these segments could possibly have uterine origin, and would thus be due to a contraction, or could alternatively be caused by artifacts.

The corresponding segments in the EHG signal were classified as artifacted or non-artifacted signals by two experts (1 bioengineer and 1 clinician) with the help of the simultaneous TOCO and IUP recordings and the previously annotated events. Segments classified as artifacted signals should correspond in time to annotated events of mother or fetal movements, except for visually identifiable abrupt variations of the biosignals which were also considered artifacted signals since this behavior does not have an uterine physiological origin, and these episodes could have been missed in the annotated events. The segments classified as contractions (non-artifacted signals) had to correspond in time to uterine pressure events as measured by IUP and TOCO. In this study a segment of signals with both contraction and artifacts is considered to be an artifacted signal. We decided to work in this manner since when an artifact is present during a contraction it affects the signal parameters which could lead to misinterpretation of the EHG characteristics associated to that contraction. Only the segments in which the classification of both experts agreed were included in the design and test of the automatic classifier. A total of 277 EHG artifacted and 422 non-artifacted signal windows were used.

### 2.3. Feature Analysis

Motion artifacts in surface myoelectric recordings come in a wide range of waveforms according to the type of motion and the subject characteristics. Besides their presence is intermittent and unpredictable [18, 19]. For example, pulse-type motion artifacts often cause abrupt variations in the potential measured on the abdominal surface, while others are associated with a considerable rise in the potential amplitude. The presence of motion artifacts also affects the power spectral density (PSD) of the signal, distributing its energy in the high frequency range [18, 20]. In this study, the following EHG signal features were proposed to detect their presence.

*Spectral Parameters.* To determine the energy distribution within the signal spectrum, the energy was calculated in certain frequency ranges [23]. Given the amplitude variations in the EHG signals obtained from the different channels and subjects during the sessions, this energy was normalized in relation to total energy. Three frequency ranges were defined to characterize energy distribution in the signal spectral domain (*E*_1_: 0.1–0.3 Hz; *E*_2_: 0.3–1 Hz; *E*_3_: 1–4 Hz):

(2) $$\begin{array}{c} E_{j}=\frac{\sum_{f_{k}=f_{0j}}^{f_{1j}}\text{PSD}\left[f_{k}\right]}{\sum_{f_{k}=0.1}^{4}\text{PSD}\left[f_{k}\right]}, \end{array}$$

where PSD [*f*_*k*_] is the bipolar signal PSD obtained from the periodogram with a Hamming window and *f*_0*j*_ and *f*_1*j*_ are the abovementioned lower and upper limits of the frequency band considered (*j* = 1, 2, 3). 

*Temporal Parameters.* As previously mentioned, EHG signals containing artifacts often present sudden large amplitude variations. This can be characterized by means of parameters such as standard deviation (*σ*_*x*_); relative amplitude (RA); kurtosis (*κ*); normalized maximum derivative in relation to standard deviation of the baseline (MD_bs_); normalized maximum derivative in relation to standard deviation of the signal under study (MD_*x*_); and the ratio between the RMS value of the segment of the signal under study and the RMS of the baseline extracted from the same channel and the same recording (*R*_RMS_):

(3) RA=max{xi}−min{xi}σx, i=1,…,N,MDbs=max{|xi−xi−1|}σbs, i=2,…,N,MDx=max{|xi−xi−1|}σx, i=2,…,N,

where *x*_*i*_ is the *i*th sample of the bipolar EHG signal, *N* is the number of samples in the window length, *σ*_*x*_ is the standard deviation of the signal under study, and *σ*_bs_ is the standard deviation of the baseline extracted from the same channel of the same recording session.

*Nonlinear Parameters.* The presence of artifacts in an EHG signal may affect the signal non-linearity properties, such as regularity or complexity of finite length time series which can be measured by the sample entropy (*E*_*n*_). This nonlinear technique seems to be an appropriate quantitative tool to measure the variability of underlying physiological mechanisms. It has been shown to discriminate between EHG signals of term and preterm deliveries [30], and it has been used for detection of eye blink artifact in multichannel EEG data [31]. We established a signal pattern dimension *m* = 3 and a pattern matches margin *r* = 0.15 to obtain the parameter sample entropy. In addition time reversibility of the surrogate time series (*T*_*r*_) was calculated. Probabilistic properties of artifacted signals are expected to be more susceptible with respect to time reversal than non-artifacted signals. The difference between the time reversibility of the original data and the surrogates was quantified as the measurement of signal non-linearity. For this the *z* score value was computed:

(4) $$\begin{array}{c} z=\frac{\left|T_{r\text{org}}-\langle T_{r\text{surr}}\rangle \right|}{\sigma_{T_{r\text{surr}}}}, \end{array}$$

where *T*_*r*org_ is the time reversibility of the original data, *T*_*r*surr_ denotes the time reversibility for the 100 computations of the surrogate time series, and *σ*_*T*_*r*surr__ is the standard deviation. The definition of the signal time reversibility and the method for generating surrogate time series is described in previous works [32].

### 2.4. Feature Selection

An important aspect in the design of a classifier is the selection of the features involved in it. The use of a single or a limited number of these could adversely affect the classifier accuracy due to lack of information. On the other hand, too many features could also give rise to an excess of information and over-training of data, which would also affect the classifier performance [33]. We opted for first determining which features contained the best information for distinguishing between EHG signals with and without artifacts and thus implemented a single-feature classifier in order to determine its individual discriminatory capacity [18]. Then the combination of features that gave maximum classifier accuracy in detecting artifacts by means of the sequential forward feature selection algorithm was found. The latter consists of an iterative process that checks whether or not the addition of a new feature will reduce classification errors and then selects the one with the least errors.

### 2.5. Design of the Classifier

In the present study, linear (LDA) and quadratic discriminant analysis (QDA); and support vector machine (SVM) classifier using RBF kernel was implemented. In order to determine the generalization capacity of the new data classifiers, in a first stage signals from ten patients were used (392 nonartifacted contractions and 253 artifacted segments). Specifically, two-fold cross-validation was used, with 50% of the data being used for training and 50% for validation [22]. In the second stage, classifiers were tested using signals from 2 additional patients (30 non artifacted contractions and 24 artifacted segments). Due to the random nature of the set of data used for training and validating, the cross-validation process was carried out 50 times to minimize bias. For each set of training data, various classifiers based on LDA and QDA and SVM (RBF kernel with optimized parameters) were implemented to distinguish between signals with and without artifacts. For each set of training data, optimal parameters for SVM classifier were carried out using the simplex method. All sets of data were then examined using these classifiers. Finally, classifier accuracy, sensitivity, specificity, positive predictive value (PPV), and negative predictive value (NPV) were analyzed and compared while using the best combination of features.

## 3. Results 

**Figure 2:**
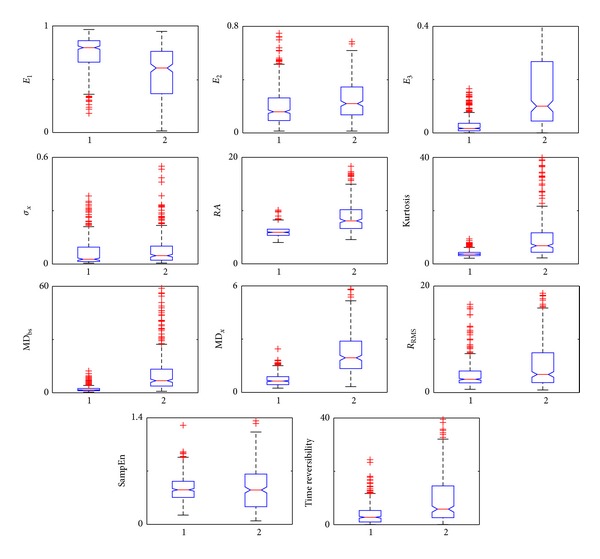
Influence of motion artifacts on EHG features. 1: non-artifacted EHG signal windows. 2: artifacted EHG signal windows. The feature *E*_3_ upper quartile value for artifacted EHG signals (Group 2) is out of scale.

Figure 2 shows a box and whisker plot of the 11 features of the EHG signals corresponding to Group 1 (no artifacts) and Group 2 (with artifacts). It can be seen that the presence of artifacts in the signal significantly raises the spectral content in the high frequency range (1–4 Hz, *E*_3_). By contrast, even though differences were found in the spectral content in the 0.1–0.3 Hz (*E*_1_) and 0.3–1 Hz (*E*_2_) frequency ranges in both groups, the distribution of these two features is completely overlapping. In the temporal parameters, the presence of artifacts is also associated with a significant rise in the values of parameters RA, *κ*, MD_bs_, and MD_*x*_. On the other hand, even though the standard deviation of the signal (*σ*_*x*_) and the R_RMS_ feature in EHG signals with artifacts tends to be higher than in signals with no artifacts, the distribution of these parameters shows considerable overlapping between both groups. As expected, the signals containing artifacts present a higher degree of nonlinear behavior as evidenced by the higher time reversibility *z*-score value, although the sample entropy in both groups is completely overlapping.

Table 1 shows the average accuracy of the single-feature classifier of the three classifiers obtained from the training and validation set of data. In general, SVM provided slightly better results than QDA, and this latter permits to achieve better accuracy than LDA. It can be seen that an accuracy higher than 75% can be obtained with the *E*_3_, RA, *κ*, MD_bs_, and MD_*x*_ features for the three classifiers. The sequential forward feature selection algorithm provided a set of 7 features as the best combination of features for both QDA and SVM, 5 of them being common for both classifiers which provide complementary information among them.  Table 2 gives the classifiers' accuracy for artifact detection in EHG signal using the best combination of features for QDA classifier, which are: *E*_3_, RA, *κ*, MD_bs_, MD_*x*_, *E*_*n*_, and *T*_*r*_. The optimal combination of specific features for LDA and SVM provided similar results to those shown in Table 2, with a difference less than 1%. It can be seen that LDA classifier presents the lowest accuracy values whereas similar results were obtained for QDA and SVM mean accuracy for the training and validation data set (92.1% and 93.3%, resp.). Nevertheless, for the test data set QDA clearly provided the highest accuracy values. Tables 3 and 4 show the values of additional prediction parameters for the training and validation and for the test data sets, respectively. Again it can be observed that LDA provides the worst results and that SVM and QDA present similar performance for the training and validation data set. In general it can be observed that the classifiers obtained higher PPV and specificity than NPV and sensitivity. This is probably due to the unbalanced database which contains a higher number of non-artifacted windows than of artifacted windows. Finally, it should be pointed out that the poorer performance of SVM in the test data set in comparison to QDA is also manifested in Table 4. This will be discussed in the next section.

**Figure 3:**
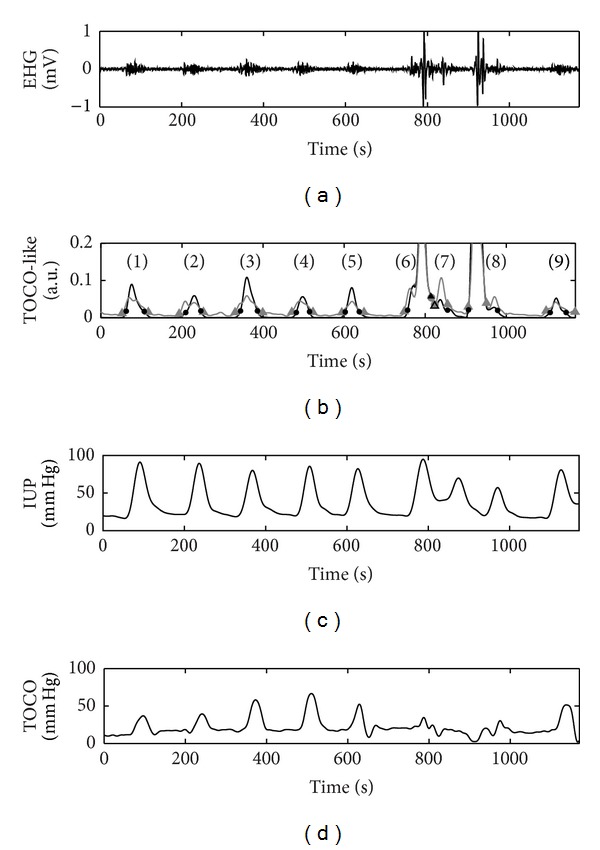
(a) EHG signal. (b) TOCO-like signal generated from EHG signal using RMS-based algorithm (grey line) and the unnormalized first statistical moment of the frequency spectrum algorithm (black line). The signal windows with amplitude significantly different from the baseline identified by the automatic contraction detector are marked by grey triangle and black point, respectively. (c)-(d) IUP and TOCO were acquired simultaneously.

**Figure 4:**
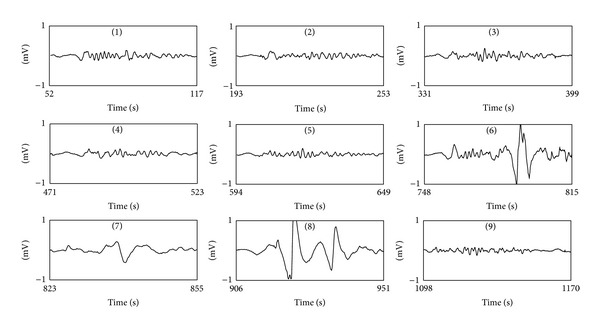
Waveform of 9 EHG signal windows identified by automatic contraction detector extracted from the recording session shown in Figure 3 using RMS-based algorithm.

Figure 3(a) shows a bipolar signal from an EHG register taken during the early stages of labor; the other three bipolar signals are not shown due to space issues.  Figure 3(b) shows the corresponding TOCO-like signals using the RMS-based algorithm (grey line) and the unnormalized first statistical moment of the frequency spectrum (black line). The automatic detector of possible contractions identified 9 signal segments with a significant rise in amplitude in relation to baseline in both TOCO-like signals. Slight differences in the onset and the end of these segments can be observed. The waveforms in these 9 signal segments are given in greater detail in Figure 4. They were later evaluated by the classifiers designed to determine whether they were associated with uterine contractions or were simply due to the presence of motion artifacts. The results suggest that the signal windows (6) and (8) identified in the two TOCO-like signals contained artifacts, while the remainder could be considered as artifact-free uterine contractions. These conclusions coincide with visual observations and the previous classification carried out by the experts. Moreover the simultaneously recorded IUP and TOCO recording corroborate this finding. In this case, contractions situated around 800 and 930 s (which coincide with windows (6) and (8), classified as containing artifacts) can be identified in the IUP. Nevertheless, these contractions cannot be correctly identified in the TOCO recording. This was possibly due to movements made by the patient at this time, which would have given rise to incorrect readings not only in the EHG recording but also in the TOCO. In such cases, even though simultaneous contractions have occurred, no robust information about the characteristics of these contractions can be obtained from either noninvasive recording.

**Figure 5:**
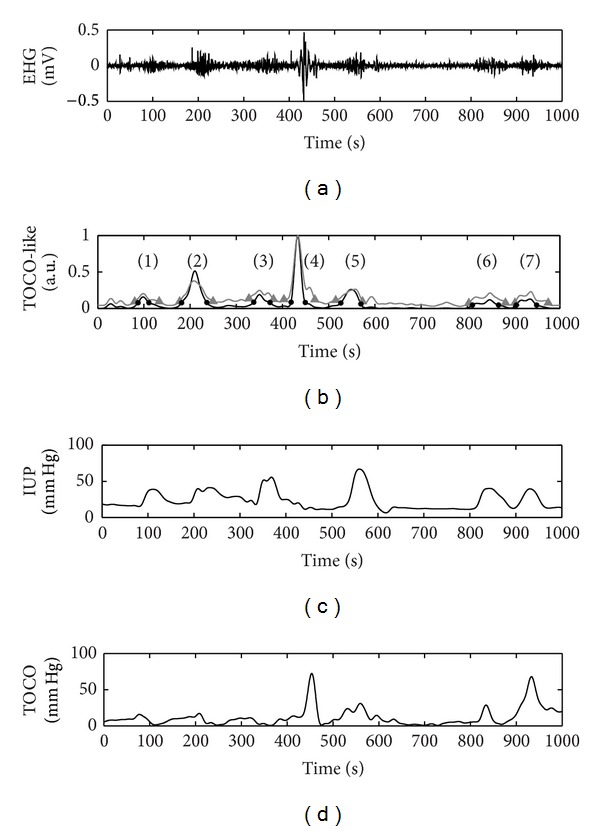
(a) EHG signal. (b) TOCO-like signal generated from EHG signal using RMS-based algorithm (grey line) and the unnormalized first statistical moment of the frequency spectrum algorithm (black line). The signal windows with amplitude significantly different from the baseline identified by the automatic contraction detector are marked by grey triangle and black point, respectively. (c)-(d) IUP and TOCO were acquired simultaneously.

Another example of the application of the algorithm designed to automatically segment and classify EHG recordings is shown in Figure 5. A total of 7 signal windows were identified with a significant increase in amplitude in both the TOCO-like signal generated using the RMS-based algorithm and that obtained from time-frequency-based algorithm, showing again some minor difference in the onset and the end of these segments. Subsequent analysis of the corresponding EHG signal segments with the classifiers showed that only the signal window (4) was associated with artifacts, while the other 6 signals windows would be of artifact-free uterine contractions. In contrast to the previous example, in this case the artifacted window does not coincide with a simultaneous uterine contraction. The comparison of the TOCO-like signal with the IUP and TOCO recordings acquired simultaneously corroborates this result. The sudden amplitude rise that occurs around the second 450 was observed in both non-invasive TOCO recording and EHG recording, but no uterine contraction was recorded by IUP recording.

## 4. Discussion

Motion artifacts detection is a common problem in bioelectrical signal analysis and is extremely challenging as their characteristics show an extremely large variability depending on the specific source, making it hard to distinguish between target signal and artifacts. Although previous works have been made in this respect [18, 20, 21, 34–36], to our knowledge this is the first one in EHG recordings. In this paper, a method for the automatic detection of motion artifacts in EHG has been proposed without the need of additional accelerometers. This method was implemented in two steps: firstly a TOCO-like signal from the EHG recording was derived and the segments with amplitude significantly different to that of the baseline were identified. Subsequently a classifier for discriminating whether this signal segment is artifacted or not was implemented.

Concerning the TOCO-like generation from EHG recording, various methods that have been proposed in the literature were implemented and compared: RMS-based approach [12, 29] and the unnormalized first statistical moment of the frequency spectrum derived from time frequency analysis [15]. It was observed that similar TOCO-like signals can be obtained from these two methods. The latter method may give a better estimation of IUP from EHG recording [15], nevertheless both methods showed similar behavior in identifying signal segments with a significant increase in TOCO-like amplitude. Only, small differences in the width of the segment were found. In this sense, if the goal is to quickly identify those signal segments with an amplitude significantly higher than that of the baseline, the RMS-based method would be preferred due to its smaller computational cost.

Moreover, in this work it has been shown that the signal features of artifacted-EHG segments differ significantly from the non-artifacted ones. Artifacted EHG segments are associated with a rise in relative amplitude, maximum derivative, and kurtosis value. These observations agree with other authors that analyzed noninvasive recordings of other myoelectric signals [18, 37]. Motion artifacts in EHG recording are also associated with a rise in relative energy between 1 Hz and 4 Hz. This is mainly due to the fact that the signal-noise ratio of EHG component decreases greatly over 1 Hz. This is the reason why several authors reduce the upper limit of signal bandwidth to 1 Hz for EHG signal feature extraction [9, 22, 28]. In addition, nonlinear parameters such as surrogate time reversibility were also tested, and clear differences were found between artifacted and non-artifacted EHG segments. Although it has been shown that the signal length has high effect on generating the surrogate data [38], we can discard this fact as the main responsible for the differences that were found since the average difference in signal length between artifacted and non-artifacted EHG signal windows was only about 6 s. Also, we tested (not shown) enlarging and reducing such difference with additional ±10 s, and the much greater values of time-reversibility parameter for the artifacted EHG segments remained; suggesting their higher nonlinearity character in comparison to non-artifacted EHG segments. It should also be noted that only monovariate features were analyzed in this work. The use of bivariate parameters associated to the correlation or synchronization between signals could also provide valuable information for describing and discriminating artifacted and non-artifacted EHG segments. This would be further studied in future work.

In the present work, the ability of the different single features for discriminating the target signal and motion artifacted signal was further analyzed. Our experimental results are in partial agreement with another study on the analysis of parameters for detecting artifacts in surface electrogastrogram recordings [18]. In this latter work, neuronal network-based classifiers were obtained with an accuracy of 94.9%, 96.2%, and 97.4% for standard deviation, high frequency energy, and maximum derivative of signals, respectively. In the present study, the accuracy obtained in nonnormalized parameters, such as the standard deviation, is about 60%, which indicates that this type of parameters has a relatively low capacity to discriminate between artifacted and non-artifacted signals. This could possibly be due to the wide variation in EHG signal amplitude between the different channels and recording sessions. By contrast, the accuracy achieved by normalized features such as *E*_3_, MD_bs_, and MD_*x*_ ranged from 76.0% to 87.6%.

On the other hand, various classifying techniques (LDA, QDA, and SVM) to distinguish the EHG signal segments with and without artifacts were compared. As it could be expected, the two nonlinear methods provided superior classifier accuracy than LDA which may be due to the fact that the features' distribution for artifacted signal and non-artifacted signal was highly overlapped. Regarding SVM and QDA, they yielded similar results for the training and validation data set. Theoretically, the SVM should provide lower generalization error [39]; however, SVM obtained significant lower accuracy values in the test data set than in the training and validation data set. Although the data set used for the design of the classifier contained more than six hundred signal windows, the data from the two additional subjects of the test set seems to have compromised the values of the support vectors of the designed SVM classifier. A database with a higher number of subjects would help to enhance the generalization capability of this classifier. Nonetheless, the results suggest that the classifier based on QDA using the best 7 features possesses a high degree of generalization for detecting artifacts in EHG signals (extendable to signals not initially included in the data base), which can therefore be considered suitable for automatic artifact detection in these signals. Furthermore, from the computational point of view, discriminant analysis is much more effective than SVM and it does not need the optimization of the classifier's configuration parameters, which is a crucial part of advanced techniques. Nevertheless, it should be highlighted that the proposed method has been tested on measurements performed during the first stage of term labor, and its feasibility for for preterm/non in labor measurements should be checked in future studies. On one hand, the interpretation of the EHG signal of pregnant patients at earlier gestational ages is more challenging due to its poorer signal-to-noise ratio, also making the detection of uterine contractions harder. On the other hand, as pregnancy progresses the uterine myoelectrical activity shifts towards higher frequencies and becomes more organized [3, 32]; therefore pregnancy contractions would also present some differences in the characteristic parameters used in this study; still they would be expected to remain different enough from those of artifacted signal windows. The inclusion of other features such as the conduction velocity [8] or the nonlinear correlation coefficient *h*2 [24] that have been shown to provide additional information in EHG interpretation could help to improve the system's performance under these circumstances.

With respect to the motion artifacts detection in bioelectrical recording, manual identification by experts based on previous knowledge about both the target signal and motion artifacts has been often used [18, 34]. Other authors consider that annotations or accelerometers [36, 40] are more objective for the detection of motion artifacts. Nevertheless the automatic identification of motion artifacts in accelerometers signal is still problematic due to its high variability, and on the other hand annotations are not absolved of the subjectivity of each patient or observer. In this work, a method based on the features of the target signal and motion artifacts was proposed and checked with annotation method. This method could be of special interest as an incentive for the use of non-invasive myoelectric techniques in clinical environments since no additional accelerometers are needed for motion artifacts detection. On the other hand, a large percentage of motion artifacted segments in EHG recording were obtained in this work. Although the subject was asked to be as quite as possible during the recording session, motion artifacts in EHG recordings are unavoidable. Moreover the occurrence of a uterine contraction may also induce movement artifacts due to abdominal deformation, due to forced respiration patterns, or due to pain. In fact, a large percentage of artifacted segments occurred during a uterine contraction that could be simultaneously identified in the IUP and TOCO recordings. This phenomenon can be observed in the segments n°6 and n°8 in Figure 3 which were associated with an amplitude rise in IUP recording. The presence of motion artifact may impair greatly in temporal and spectral parameters, and also on the measurement of conduction velocity and direction. Thus misinterpretation of the results may occur. For this reason, in this work it was preferred to classify such cases as artifacted signals not suitable for the characterization of uterine contractile behaviour.

Finally it should be noticeable that EHG recording is not only contaminated by motion artifacts but also by a set of physiological interferences, such as fetal and maternal ECG activity and respiration. Regarding the possible effects of such interferences in the proposed algorithm, ECG interference is partially cancelled in bipolar EHG recording, its energy content is mostly distributed over 1 Hz, and it is almost constant throughout the recording sessions. Therefore the proposed algorithm would not be very sensitive to this interference. Nevertheless, several techniques have been proposed for removing ECG from EHG recordings and could be used prior to applying the presented method [17, 41–43]. The respiration interference is mainly distributed within 0.20 and 0.34 Hz, which is partially overlapped with uterine electrical activity. For this reason, many authors prefer to analyze the EHG signal over 0.34 Hz [3, 22, 23, 28], although it has been shown that EHG component distributes its energy from 0.1 Hz [17, 26]. This respiration interference usually happens during a large period of time and does not suffer large variations in amplitude by contrast to uterine electrical activity, and therefore it would not be detected as possible contraction segments in our algorithm.

With respect to the potential use of EHG recordings and the proposed method in everyday clinical practice, although clinical staff is not accustomed to EHG recordings for monitoring uterine contraction, they are familiar to other bioelectrical recordings such as electrocardiogram or electroencephalogram. Therefore we consider that the progressive implementation of these methods would not be distressing. It would undoubtedly require a training period for the clinical staff to adapt to and learn about the electrode arrangement for the recording and electrode and bioamplifier wiring and handling. In this context, the TOCO-like signal generation with which clinicians are accustomed will also facilitate the introduction of this technique in clinical practice. Moreover the proposed algorithms do not require a high computational cost, and, from the user point of view, the application could be considered to work on real-time. The proposed method would greatly facilitate the task of segmenting recording sessions and evaluating uterine contractions based on the EHG recording. After having correctly identified the contractions, delivery room staff could be provided with relevant information on their efficiency, such as duration, frequency, signal amplitude, dominant frequency of the EHG signal, and the energy distribution in the spectral domain, among others [2, 5, 7–9, 22, 30].

## 5. Conclusion

The experimental results show that the most important features for detecting artifacts in EHG signals are *E*_3_, RA, *κ*, MD_bs_, MD_*x*_, sample entropy, and surrogate time reversibility. The proposed classifier, based on QDA with these features, can be used for the automatic detection of artifacts in the EHG recording, reaching a classification accuracy of 92.2%. This classifier, jointly with the proposed TOCO-like signal generation and analysis algorithms, provide a tool for the automatic detection and segmentation of uterine contractions, distinguishing them from possible artifacts. This technique could therefore be a valuable aid to the analysis of surface EHG recordings and could be used by clinical staff to extract additional information from the habitually used TOCO recordings.

## Figures and Tables

**Table 1:** Mean accuracy (%) of classifiers using a single feature for detecting motion artifacts (training and validation data set, 392 nonartifacted contractions versus 253 artifacted segments).

	*E*_1_	*E*_2_	*E*_3_	*σ*_*x*_	RA	*κ*	MD_bs_	MD_*x*_	*R*_rms_	*E*_*n*_	*T*_*r*_
LDA	70.6	61.2	78.5	55.7	80.9	76.9	76.0	85.3	66.2	54.6	67.4
QDA	71.4	61.6	78.8	59.0	80.8	78.7	78.1	86.2	65.5	69.6	69.5
SVM	70.9	62.6	82.1	63.6	80.9	79.9	83.4	87.6	66.0	70.9	69.3

**Table 2:** Comparison of the classifiers' accuracy using the best combination of features for motion artifact detection in EHG signals.

	LDA	QDA	SVM
Training + validation	86.1 ± 0.8%	92.1 ± 0.3%	93.3 ± 0.6%
Test	79.4 ± 3.5%	92.2 ± 1.8%	83.6 ± 3.5%

**Table 3:** Comparison of the classifiers' performance for the training and validation set of data (392 nonartifacted contractions versus 253 artifacted segments).

*N* = 645	Sensitivity	Specificity	PPV	NPV
LDA	69.4%	97.0%	93.7%	83.1%
QDA	84.3%	97.0%	94.8%	90.6%
SVM	87.1%	97.3%	95.5%	92.1%

**Table 4:** Comparison of the classifiers' performance for the test data set (30 nonartifacted contractions versus 24 artifacted segments).

*N* = 54	Sensitivity	Specificity	PPV	NPV
LDA	58.0%	96.5%	92.9%	74.2%
QDA	90.7%	93.5%	91.7%	92.6%
SVM	77.7%	88.4%	84.3%	83.2%
